# Organically Linking Green Development and Ecological Environment Protection in Poyang Lake, China Using a Social-Ecological System (SES) Framework

**DOI:** 10.3390/ijerph18052572

**Published:** 2021-03-04

**Authors:** Ji Feng, Zheng Zhao, Yali Wen, Yilei Hou

**Affiliations:** 1 College of Economics and Management, Beijing Forestry University, Beijing 100083, China; fengji2009@bjfu.edu.cn; 2 College of Tourism, Shanghai Normal University, Shanghai 200234, China; zzshnu@shnu.edu.cn

**Keywords:** social-ecological system framework, Poyang Lake area, wetland ecosystem, green development

## Abstract

Wetlands are unique ecosystems formed by the interaction between land and water on Earth. Poyang Lake, which is a part of China’s largest freshwater lake wetland, is well known for its ecological and economic importance. This study uses a social-ecological system (SES) framework that integrates watershed and human activities; we used action scenarios to analyse the influencing factors, solutions, and feedback mechanisms involved in the SES framework. We chose Nanchang, Jiujiang, and Shangrao in the Poyang Lake wetlands of the Jiangxi province as the study areas to provide a problem-oriented analytical strategy for the organic interface between ecological conservation and green development. The key issues indicate that the Poyang Lake region faces multiple problems, such as ecosystem structural changes and environmental pollution, caused by anthropological activities, inefficient implementation of conservation policies, and insufficient funding for pollution prevention and control. Our findings provide a systematic solution for major conservation and development issues in the Poyang Lake region and are adapted to the characteristics of the lake. We also provide a theoretical reference and direction for the implementation of green development and modernisation of ecological governance in the Great Lakes basin in China.

## 1. Introduction

Ecological and environmental problems are becoming increasingly prominent worldwide, posing a serious threat to the sustainable development of human society. Protection of the ecological environment has not only become an urgent social need but a common challenge and everyone’s responsibility. In 1972, the Human Environment Declaration, which was adopted by the United Nations Conference on the Human Environment, became the policy of action for the ecological and environmental protection in several countries. In this conference, for the first time, environmental problems were considered a global problem. The search for solutions to environmental pollution problems related to industrial production has become the core of green development. In 2008, the United Nations urged countries around the world to actively implement the “Green Economy” and “Green New Deal”. In 2015, the United Nations Sustainable Development Goals (SDGs) for 2030 were launched, forming the basic framework for a total of 17 goals. Protecting, restoring, and promoting the sustainable use of terrestrial ecosystems are key elements of SDGs 15—the service functions of the ecosystem and its sustainability have always been emphasised and valued. The connotation of green development ideas is constantly evolving and improving.

China is also facing serious ecological challenges that may hinder its future development. China’s ecological function and fragile ecological zones mostly overlap with less developed regions, and they have a prominent ecological safety barrier function [1]. At the same time, these areas impose restrictions on the use of natural resources, which is an important cause of the conflict between conservation and development. In addition, the Chinese government has made a commitment to the SDGs by organically integrating the sustainable development agenda with China’s national medium and long-term development plan and have set out details and programmes for specific goals.

Therefore, in the context of the 2030 sustainable development goals, it is very relevant to study the relationship between ecological protection and green development. Poyang Lake provides a good case study for the research. At present, Poyang Lake is facing the same “development paradox” dilemma as other developing countries in the world; the ecological environment and fluctuation of ecological laws have been critically damaged in the process of economic development and income growth [2] and are troubled by the serious contradiction between protection and development. In terms of economic development, continuous population growth and irrational economic development patterns have exacerbated the degradation of the wetland ecosystem. In terms of ecological environment, the lack of policies related to wetland protection has caused frequent occurrences of tragedy of the commons in the Poyang Lake area. Poyang Lake is undergoing a period of overlapping and advancing economic development and ecological protection and converging history [3]. Therefore, realising the organic link between green development and ecological environmental protection in the Poyang Lake region has become an urgent and significant problem in reversing the decline of the ecosystem service function in the Yangtze River basin. This is also crucial for helping the ecologically fragile area of Poyang Lake to deal with its ecological management crisis and to realise the coordination between environmental protection and economic development.

We reviewed previous studies extensively and found that scholars have analysed the connotation of green development from the perspective of the relationship between the natural environment and human, social, and economic development. Charest analysed the relationship between green development and water resources, human health, and economic development during the study of the Canadian Green Plan and reported that a connotation of green development is the coordination of natural environment and human activities [4]. There are also several scholars who directly studied the embodiment of green development in the economy, expanded the connotation of green development concept from the perspective of the green economy, and analysed the connotation and mechanism of green economy from the perspective of environmentalism. “Green” is the symbol of healthy development of the environment and a green economy is the product of environmental protection. When discussing the development path of the green economy, many scholars have analysed the operating mechanism and internal requirements of the green economy and interpreted the characteristics of changes in economic development from red to green environmental management policies [5,6,7]. In addition, scholars have also analysed the basic characteristics and developmental requirements of the green industrial revolution and discussed the institutional thinking about the green economy; they have studied the impact of the green economic structure on the world using specific countries as examples and the relationship between green economy and smart growth under neoliberalism [8,9,10]. In the field of wetland conservation, in recent years, researchers have focused more on enhancing sustainable watershed management by developing policies to implement ecological restoration, assessing the impacts of conservation projects on wetland ecosystems using a conceptual model to define socio-economic vulnerability, and considering different stakeholder perspectives to enhance sustainable watershed management [11,12,13,14,15]. This provides an opportunity for organisations such as the Society of Wetland Scientists to raise the profile of wetlands and to initiate a discussion on how to respond to and change direction of the destructive development trajectory that led to wetland loss and degradation [16].

Additionally, the field of social sciences has begun to focus on ideological connotations, policy design, and adaptive governance to achieve green development of regional complex ecosystems. For example, scholars compared the differences and relationships between green and sustainable development and analysed the function and mechanism of green development. They believe that green development is the effective unity of the economic, social, and natural systems. This type of development proposition not only satisfies people’s material and cultural needs but also their ecological and environmental needs, and it goes beyond the proposition of sustainable development that focuses only on economic growth [17,18]. Ecological and environmental monitoring and watershed management can be implemented through the development of watershed regulations and compensation policies. The development of wetland ecotourism can effectively protect the resources environment and promote local social and economic sustainable development [19,20]. Green development in China has distinctive Chinese characteristics; it not only draws upon the characteristics of international green thinking, but also includes the national concept of development thinking that considers lucid waters and lush mountains as invaluable assets of China.

Traditional economic growth theory believes that the factors affecting economic growth are land, manpower, currency capital accumulation, economies of scale, technological progress, and human capital growth. In the short term, these factors affect the speed and quality of economic growth [21]. However, these studies show that there is still a lack of systematic and in-depth research on the internal relationship, feasibility, existing problems, and connection paths of the organic link between ecological environmental protection and green development; these issues are the key to the effective implementation of ecological civilization. As people’s understanding of human–environment interactions continued to deepen, they gradually realised the shortcomings of evaluation of the natural ecosystem only and focused on the comprehensive study of human–environment interactions. There is an increasing need to consider the integrated system of social environment and natural ecological environment as an important factor in the exploration and analysis of development issues [22]. In recent years, the social-ecological system analysis method and its framework construction have become topics of great interest in the field of international natural resource governance and sustainable development. The framework focuses on multiple interactions between natural ecosystems and social economic systems and explores the mechanism and evolution of this interaction. By solving multidimensional, multiscale, and multistage comprehensive problems, the framework provides a variety of governance methods and policy tools that are in line with the local reality of the sustainable development of the region [23,24,25,26,27,28]. Therefore, in this study, we aimed to construct a wetland social-ecological system framework in Section 2. In Section 3, we conduct an assessment to analyse the state, changes, and intrinsic relationships in the ecological and economic systems of Poyang Lake. In Section 4, we systematically analyse the internal problems and influencing factors in the process of convergence between green development and ecological environment protection, adopt ecological protection engineering and green development planning at the methodological level, and explore a practical avenue to realise the organic interdisciplinary convergence of the social system and the ecosystem in the lake area. Finally, in Section 5, conclusions and research recommendations are provided, focusing on the practical implications of our study results for advancing the organic integration of ecological protection and green development in the Poyang Lake area.

We hope that these results will provide a new analytical perspective for identifying and evaluating the problems involved in the regional ecological protection and socio-economic coordination. We also hope that our study aids in finding a feasible problem-oriented solution to the dilemma of coordinated social-ecological system development in the Poyang Lake region, providing effective theoretical support for organically linking ecological environmental protection and green development of wetland ecosystems in the great lake basins in China, and also providing the response strategies of China for achieving sustainable development on SDG.

## 2. Materials and Methods

### 2.1. Study Area

**Figure 1:**
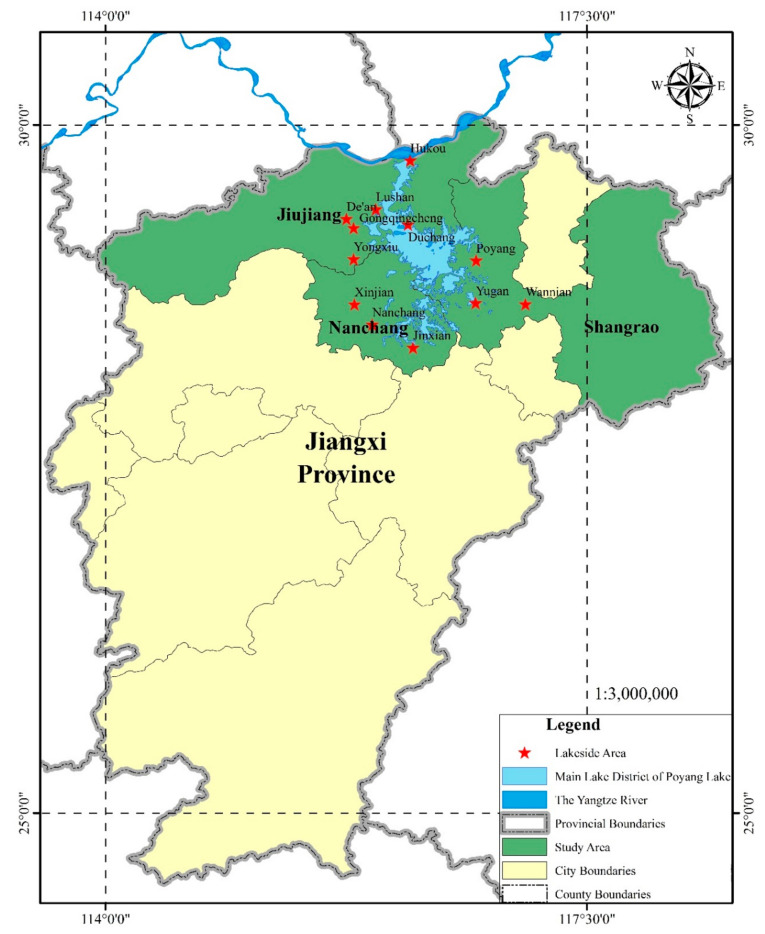
Study area.

Poyang Lake, north of Jiangxi province (115°49′–116°46′ E, 28°24′–29°46′ N), which is the largest permanent freshwater lake wetland in China, is located on the south bank of the Yangtze River (Figure 1). It is located in the East Asian monsoon region and is characterised by a mild climate and abundant rainfall. The total area of Poyang Lake is 3519 km^2^, of which the wetland area is 3464 km^2^. There are 231 wetland patches, accounting for 95.21% of the lake wetland area in the Jiangxi province. Poyang Lake has a very important strategic position in the socio-economic development and ecological security pattern of the Yangtze River basin in China and is an important wetland with global influence.

We selected Nanchang, Shangrao, and Jiujiang near Poyang Lake in Jiangxi province as the case study sites. This region is an ecological and economic system according to the natural geographic area of Poyang Lake, which is composed of land and water ecosystem, and network-type economic region of Poyang Lake; the lake is at the centre. These sites are located at the intersection of the Yangtze River Economic Belt (Yangtze River Economic Belt covers 11 provinces and cities including Shanghai, Jiangsu, Zhejiang, Anhui, Jiangxi, Hubei, Hunan, Chongqing, Sichuan, Yunnan, and Guizhou, with an area of 2,052,300 km^2^, accounting for 21.4 % of the total area and more than 40% of the population and gross domestic product (GDP) of the country) and the Economic Cooperation Zone of Jingjiu (economic cooperation zone of Jingjiu currently has 17 member cities, containing seven provinces and 127 counties (cities and districts), with a land area of 200,000 km^2^ and a total population of 80 million), which is adjacent to the Wuhan Metropolitan Area (Wuhan Metropolitan Area (WMA), also known as Wuhan “1+8” city circle, is a city cluster with Wuhan (largest city in central China) as the centre, covering eight large and medium-sized cities around Huangshi, Ezhou, Huanggang, Xiaogan, Xianning, Xiantao, Tianmen, and Qianjiang; WMA is the largest city cluster in central China), Changsha–Zhuzhou–Xiangtan city group (Changsha–Zhuzhou–Xiangtan city group, located in the east-central part of Hunan province, China, is an important part of the city cluster in the middle reaches of the Yangtze River. It includes Changsha, Zhuzhou, and Xiangtan and is the core growth pole of the economic development of Hunan province), and Wanjiang city Belt (Wanjiang city belt includes the entire territory of eight prefecture-level cities—namely, Hefei, Wuhu, Ma’anshan, Anqing, Chuzhou, Chizhou, Tongling, and Xuancheng, as well as the Jin’an district and Shucheng county of Lu’an city; it is the only regional development plan in China so far that focuses on the theme of industrial relocation). The location is the direct hinterland of several major economic plates located along the southeast coast of China and is becoming an important growth pole in central China.

### 2.2. Data Collection

From the perspective of social-ecological analysis, the degradation of the Poyang Lake wetland is rooted in the changes in the wetland ecosystem caused by population, economy, politics, science and technology, and culture, thereby affecting the wellbeing of the residents of the lake area. The development of the lake area is restricted by various conditions such as natural conditions, social conditions, policies, funds, and the ability of grassroots organisations [29]. Therefore, most of the problems related to ecological environment protection and green development can be analysed as multilevel collective actions and coupling relationships between the socio-economic system and the ecosystem in the lake area. With reference to the key attributes of each group of variables in Ostrom’s existing studies, we split the eight primary groups of variables described above into several secondary variables (or explanatory variables) [30,31], so that the social-ecological system (SES) framework can become a “diagnostic tool” that can identify the organic integration of conservation and development to form the Poyang Lake Ecological Protection and Green Development Organic Interface Analysis Variable Table (Table 1).

One of the important characteristics of the SES framework is its decomposability. Researchers can decompose the subsystems and variables in the framework horizontally or vertically according to the SES. In this study, RS refers to the main lake area of Poyang Lake, RU refers to the wetland ecosystem, GS refers to the institutional arrangements and systems related to various protection and development projects from the central government to the Jiangxi province-level design and policy system. “A” refers to individuals or organisations related to the protection and utilisation of wetlands, such as the government, society, public, and residents of the lake area; S and related ECO refer to the location of the social, economic, climate, geographical, and other background factors of the case at the city-scale. Table 1 presents the second-level variables of the SES framework. These variables provide diversified choices for action problems in different scenarios. Based on the data obtained from the field investigation and owing to the need of comparative analysis of the cases, we selected relevant variables for specific analysis, expanded a few secondary variables of the SES framework, and focused on the investigation of heterogeneous variables between different cities. Part of the research data comes from field research and the other part comes from the analysis of existing literature and statistics (see Appendix A for data source).

### 2.3. Social-Ecological System Framework (SES)

In the 1980s, many scholars pointed out that the reasons behind resource and environmental problems are “complex system problems” and these reasons are diverse, scattered, and complex [32,33]. In recent years, an increasing number of studies have shown that the interactions between human society and ecosystems are complex [23,25] and not a simple binary opposition. Ostrom proposed the social ecosystem (SES) framework that enables researchers to study the role of variables in complex social-ecological systems in greater depth and to diagnose key problems, analyse influences, set action scenarios, and assess the impact of outcomes [27]. The SES framework has been improved by theoretical researchers; the theory and analytical methods have evolved primarily for testing the effectiveness of community management of natural resources, such as fisheries and forests. However, the framework is still under development [30].

**Figure 2:**
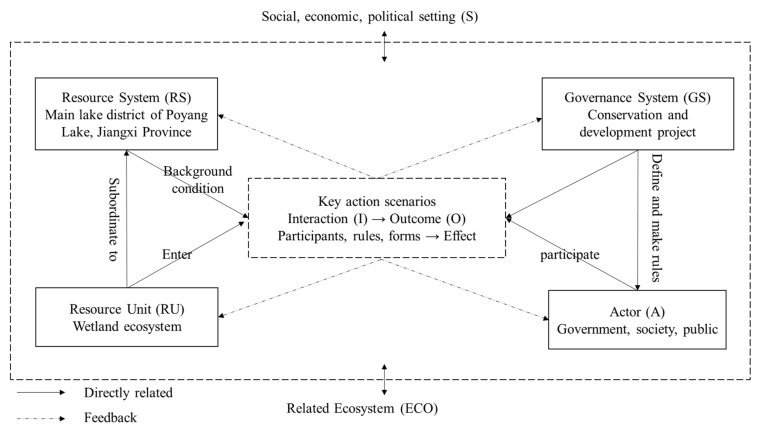
Framework of social-ecosystem system (SES) analysis.

The SES framework is a general analysis framework composed of multiple levels of variables. We have adapted the analytical framework for the Poyang Lake area taking into account existing studies by Ostrom. Figure 2 presents the first-level variables of the SES framework. In the first level of the SES framework, four subsystems (resource system (RS), resource unit (RU), governance system (GS), and actor (A)) affect the interaction (I) and the outcome (O) of the action in the action scenario. The interaction (I) and outcome (O) combine the human system and the resource system, which constitutes the key action scenario in natural resource governance [30,34]. At the same time, the interaction and outcome of all variables are affected by two subsystems—namely, two macro-variable groups of the social, economic, political setting (S) and related ecosystem (ECO).

The construction of the SES framework includes two main parts: one is to determine the problems in the ecosystem, and the other is to decide the corresponding action scenarios [35]. We have considered a comprehensive range of possible research methods, but through a comparison of relevant research literature, we found that qualitative comparative analysis was more appropriate for the problem. The quantitative research was not suitable due to the limitations of the indicator system and the amount of data [36,37,38], so we ultimately made our choice. The analytical steps are inspired by Lelie and Brondizio [34,38], who used the SES framework to operationalise the research and/or collective action problem.

**Figure 3:**
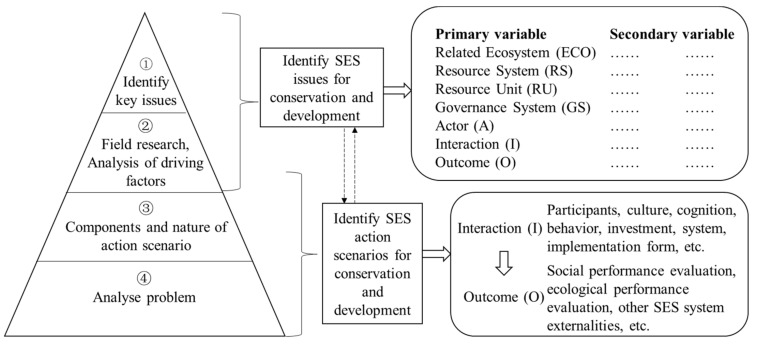
Problem-oriented social-ecological system (SES) framework setup.

As shown in Figure 3, four general steps are suggested to guide the analysis of Poyang Lake regions as SESs. Each part of the framework and its respective components are described in more detail in the following. The first step is to identify the priority problems to be diagnosed and studied in a given area. The second step should be carried out in a comprehensive way. The aim of it is to recognise the nature of the problem and understand the potential SES boundaries required by the problem through field research. As shown in Figure 3, for the second step, according to the relevant situation of the problem, we can use the seven types of coupling relationships described above to define the interaction between ecological conservation and green development issues. This interactive approach to defining issues and drivers should evolve with the change of the factors, locations and stakeholders involved. The third step is to define the key action scenario, i.e., the composition and nature of the particular issue or problem to be studied. Action scenarios can be defined at a particular level, but are always influenced by action scenarios at other levels. That is, at a particular level, there are actors and interest groups, their microbehaviours and ones influenced by informal rules, all of which influence a macro action scenario. The fourth step should focus on analysing the relevant outcomes and interactions that influence the action scenario. Action scenarios are characterised by interaction patterns within actors and with the resource units they draw on. These interaction patterns will produce social, economic and ecological outcomes. The SES framework is used on the premise that action scenarios for ecological conservation and green development issues are dynamic and evolving, where new alternatives and actions are generated to address particular issues, thus influencing the system as a whole. These outcomes should be evaluated against selected evaluation criteria, including efficiency and sustainability of resource use, distributional equity, social legitimacy, participation, accountability, financial equity, adaptability, and resilience to shocks, among other things [38].

We first determined the practical problems of ecological environmental protection and green development; then, we conducted an on-site investigation and analysis of the influencing factors of their organic linking. Second, we determined the protection and development of SES action scenarios, including the analysis of the component and nature of the action scenarios, to provide a basis for the analysis of the problems and the evaluation results. The key action scenarios mentioned here are the problems that need to be identified and the measures to be taken [39]. Each action scenario should include actors and stakeholders because their cognition, attitudes, and behaviours, and the degree of information obtained impacts a specific action scenario.

It can be seen that the process of solving development problems by protecting the ecological environment is complicated. We believe that developing ecological protection awareness among the actors will influence green development at the macro level by triggering changes in specific factors in the social ecosystem at the micro level. This requires an analysis of the complex relationships among the many variables contained in the SES of a specific area. Based on the SES framework, we combined the unique ecological protection and green development scenarios of the Poyang Lake area to conduct a social-ecological coupling analysis and develop a specific research idea and analysis logic.

Regarding the ecological system, the ecological environmental protection of Poyang Lake should not only consider the hydrological situation but also pay attention to the environmental pollution and water environment quality of the lakeside area, biological resources and biodiversity of wetlands in the core area of the lake, and the changes in the ecology of the lake. Hydrological conditions and air quality are primarily affected by the surrounding industrial planning, supporting infrastructure construction, and other factors, while the soil environment is affected by fertiliser application and irrigation methods. Biological resources include animals, plants, and microorganisms, which are mainly affected by human interference and environmental conditions [40].

For the social system, the socio-economic situation and governance system of Poyang Lake are the primary objects of analysis. Changes in the population, industrial economy, infrastructure, and social culture of Poyang Lake will have a direct impact on the regional ecological environment. Increasing population, rapid industrial development, and incomplete infrastructure challenge the carrying capacity of natural resources and threaten the ecological environment.

In November 2017, we identified the study site through interviews with the head of the Wildlife Conservation Authority of Jiangxi province. From 2018 to 2019, we conducted four interviews and surveys with staff from relevant management agencies in each of these three cities. We chose Poyang Lake National Nature Reserve, Nanji Wetland National Nature Reserve and Duchang Migratory Bird Provincial Nature Reserve as the sites for our field research and spoke with staff and some villagers who have extensive experience in conservation work. These key informants are frontline participants in the management and conservation of Poyang Lake wetlands and are actively involved in coordinating the relationship between economic development and the ecological environment. They could provide information on the status, history and causes of change of the wetlands of Poyang Lake, as well as the attitudes and behaviour of villagers. In the course of our research, we also collected statistical yearbooks from 2010 to 2019 for the cities of Nanchang, Jiujiang and Shangrao through library access and online information.

## 3. Results

### 3.1. Current Situation of Wetlands in Poyang Lake Region

#### 3.1.1. Poyang Lake Wetland Ecosystem (ECO)

Jiangxi province has a typical humid subtropical monsoon climate (Table 2). Over the past 50 years, the average water level of Poyang Lake has shown a slight downward trend. According to the statistics, the average water level of Jiangxi province in the 2000s was significantly lower in September–November than in other years, especially in 2006, 2009 and 2011 (a drop of about 5 m compared to the multiyear average). The high volume of water flowing out of the lake to downstream rivers due to sand mining in the riverbed affects the amount of precipitation to evapotranspiration in the basin and the relationship between the river and lake. The average annual siltation of sediment in the 1990s was 544.2 × 10^4^ t; in 2003, this dropped to the lowest value with a net loss of sediment of 1246.3 × 10^4^ t. In the 2010s the average annual loss of sediment was 388.8 × 10^4^ t [41]. Additionally, the cyclical climate change, i.e., the change in the cycle of dry and wet climates in the Yangtze River basin, has become prominent as well. These changes have led to a clear trend of frequent changes in water regime in Poyang Lake since 2006; however, its influence on the succession of wetland vegetation community structures is not obvious. For example, the number of phytoplankton species remained largely unchanged, and the average annual density is 10.7 × 10^4^ ind./L, an increase of 523 % [41], but the average biomass of phytoplankton decreased, and the number of large phytoplankton decreased, showing a trend of miniaturisation.

Lake wetlands absorb water into mudflats when it rains; once the water level drops, the water absorbed by swamps and peat wetlands is slowly released. The total storage capacity of Poyang Lake is 24 billion cubic metres (Data source: 2015 Poyang Lake scientific research report). The sponge effect of wetlands in absorbing floodwater becomes more obvious upstream of the river ecosystem. Analysed from another perspective, Poyang Lake also ensures the buffer absorption of floods in the Yangtze River basin. The dry and wet alternation of the Yangtze River basin has the main period of 6 years and 20 years hypoperiod (https://www.thepaper.cn/newsDetail_forward_8751727 (accessed on 6 August 2020)). During wet periods, there can be large floods, such as in 1995, 1996, 1998, 2000 and 2020. When floods occur, Poyang Lake plays a buffering and absorbing role to ensure that the floods are prevented here and avoid directly hitting downstream. According to the ANOVA results, the 12-month average water level of Poyang Lake from 2010s and 2000s shows a statistically significant changes (*p* < 0.01) in the concentrated rise in May and the rapid fall in October (Data source: 2015 Poyang Lake scientific research report). The annual flooding period occurs in this period, the Anhui and Jiangsu provinces thereby suffered. There are fewer lake wetlands in these areas, so there is no place for the flood water to go, and it could only directly damage the local residents. However, due to the absorbing function of Poyang Lake, the loss of life and property in these provinces reduced a lot. Thus, protecting the complete Poyang Lake wetland ecosystem is very important as it plays critical role during floods in the Yangtze River. It is a green “infrastructure” that solves a series of ecological problems.

#### 3.1.2. Social, Economic, and Political Setting (S) of Poyang Lake Wetland

Compared with other economically developed regions in China, Jiangxi province has a weak comprehensive economic strength and weak regional competitiveness. Twenty-five of the poverty-stricken counties in the province are key old districts and counties (http://www.gov.cn/xinwen/2020-04/27/content_5506419.htm (accessed on 27 April 2020)). At the end of 2019, the population that faces poverty decreased from 3.46 million at the end of 2013 to 96,000 according to the current standards of the province, and the poverty incidence dropped from 9.21% to 0.27% in the same time period. Thus, the overall regional poverty rate decreased, which led to new changes in the rural human settlement environment. From the perspective of economic development, the economies of Nanchang and Jiujiang, along with the lake counties located in the west of the ring lake, have become stronger, while the overall development level of the vast area to the east of the ring lake is lagging behind compared to the west (Table 3).

From the perspective of political factors, at present, the central government and Jiangxi provincial government attach great importance to the organic linking between green development and the ecological environmental protection in the Poyang Lake area. Through major ecological projects, ecological compensation and development of ecological industries, and other ecological poverty alleviation measures, national nature reserves have been established in Nanchang and Jiujiang; the employment of bird guards, forest rangers, and the policy of “work instead of support” has been adopted to stimulate the internal motivation of disadvantaged groups. The burden of pollution related to economic development often falls disproportionately on vulnerable groups—the problem of drinking water safety owing to wetland pollution may be even more serious for these groups.

#### 3.1.3. Poyang Lake Wetland Resource System

The wetland resources in Jiangxi province are very rich and have significant local and global influence. Poyang Lake, which is the largest freshwater lake in China, along with five major rivers—Laijiang, Fuhe, Xinjiang, Raohe, Xiuhe—and their tributaries, form the complete water system network of the entire province. From the perspective of RSs, three cities in the Poyang Lake district have similarities (Table 4). More importantly, Poyang Lake is located on the south bank of the middle reaches of the Yangtze River. The lake plays an important role in regulating the floods in the Yangtze River and maintaining the ecological environment of the river. It is regarded as an internationally important wetland by the World Wide Fund for Nature and is an ideal habitat and foraging place for winter migratory birds. It is also a world-famous protected and viewing area for rare birds [42] and is a ground for biodiversity conservation.

Poyang Lake is the spawning and growing ground for high-demand fishes in the Yangtze River and is an important fishery production base in the Jiangxi province; it is also the largest freshwater fishery resource bank in the province. Jiujiang has the most wetland resources, Shangrao has the most fish farming areas, and Nanchang has the highest fish farming aquaculture yield. As an important indicator of biodiversity conservation, the monitoring results of migratory birds have shown that, since 2003, the populations of 10 key protected species, including the Siberian Crane and the Oriental White Stork, have a steady upward trend (according to the field research and interview of the project team in Poyang Lake National Nature Reserve). The unreasonable prolonged use of wetland resources has not caused a significant reduction in the population of key protected species; however, it has caused changes in the structure of the ecosystem and degradation of ecological service functions.

#### 3.1.4. Poyang Lake Wetland Governance System (GS)

The goal of ecological environmental protection is not only green development, but also attracting investments in leading industries with ecological resources and increasing the wellbeing of the population with an improved environment. It also provides the foundation for a harmonious symbiosis virtuous circle for the development of industries, talents, and culture. For the wetland ecological environmental protection governance system, we primarily identified the implementation of a series of ecological protection projects and measures at the state and local scales (Table 5).

With the continuous progress of urbanisation in Jiangxi province, the socio-economic structure of the lake area has been altered. The continuous flow of urban and rural populations and the continuous differentiation of peasant groups have gradually weakened the concept of rural production and community living in the Poyang Lake district. In the short term, special stages of development can be achieved through unconventional methods by mobilising resources to a centralised organisation to achieve rapid development, so that the problem of diminished capacity for collective action is not fully apparent. However, green development is long-term and of a complex nature. The decline in governance capacity is not only insufficient for poverty alleviation but also ineffective at adequate resource mobilisation for the completion of green development in the Poyang Lake area. In particular, the implementation of the precise assistance policy has provided a disproportionate amount of assistance to the poor, leading to a disruption of the balance of interests within existing communities in some areas [43].

### 3.2. Changes in the Ecological and Social System of Wetlands in Poyang Lake

**Figure 4:**
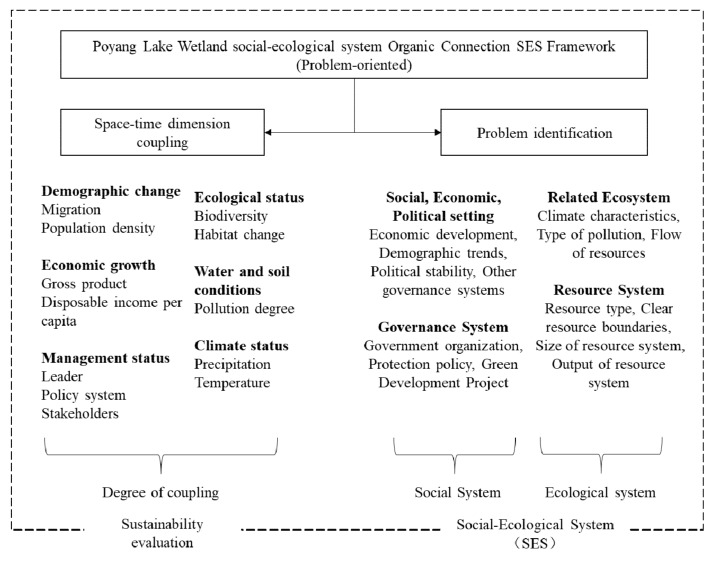
Problem-oriented social-ecological system (SES) dynamic analysis framework.

There are serious conflicts between protection and development in this area, relating to water ecological environment, soil erosion, soil pollution, biodiversity, and human–bird competition for plant resources, all of which need to be resolved to realise the green development of Poyang Lake. For this reason, it is first necessary to adopt certain standards at the macro- and microscales to identify and diagnose problems in the natural, economic, and social element systems in the basin. With reference to existing research results [13,14], we provide an SES framework for the dynamic analysis of green development and ecological protection of the Poyang Lake area in the Jiangxi province, as shown in Figure 4.

Between the ten years from 2009 and 2017, the economic situations in Nanchang, Jiujiang and Shangrao grew significantly, while the total emissions of the industrial wastewater, gas and residue were declining. The total population of each municipality is increasing and the rural population is decreasing (data compiled according to “Jiangxi Statistical Yearbook 2018” and “Jiangxi Statistical Yearbook 2010”). The persistent dryness of Poyang Lake has caused water supply and irrigation crises for the 12.4 million inhabitants of the region [44].

### 3.3. Inter-Relationships in the Ecological and Social System of Wetlands in Poyang Lake

**Figure 5:**
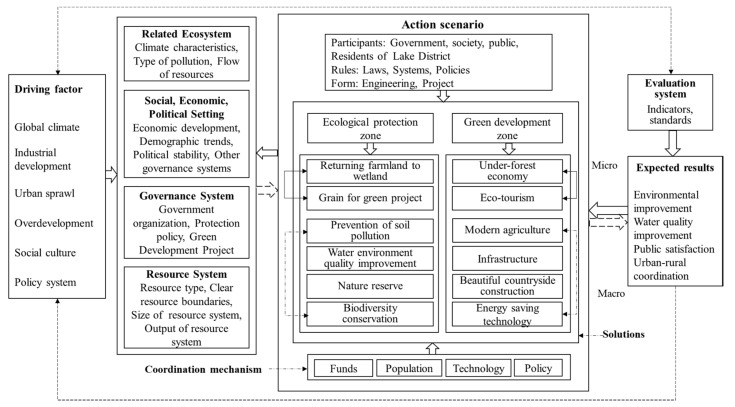
Action scenario setting based on social-ecological system (SES) framework.

To integrate green development and ecological protection organically, it is necessary to design relevant indicators based on the SES action scenario framework mentioned above [33,45]. At the macroscale, we first divided the area for protection and development, setting the improvement of the regional social, economic, and ecological coupling coordination as the main goal, and determined the priority protection areas that have important values for maintaining the ecological security and social economy of Poyang Lake. Then, we focused on the comprehensive social and ecological benefits to promote the green development of the lake area. At the microscale, we paid more attention to the residents of the lake area through the identification of residents’ protection behaviours and development willingness and developed a targeted development model. It is important to note that the eco-environmental protection and green development at the macro- and microscales should be implemented simultaneously to ensure that every aspect is effective and coordinated, and a dynamic feedback mechanism is formed during the implementation process; continuous monitoring and evaluation should be conducted in the time dimension, and a dynamic feedback mechanism should be formed during the implementation process. We set the driving factors, solutions, participants, coordination mechanism, expected results, and feedback mechanism of natural resource dependence involved in the SES framework (of ecological environmental protection and green development in the Poyang Lake area) and conducted a social-ecological system analysis of the study area (Figure 5).

We evaluated the two dimensions of social economy and ecological environment. Socio-economic indicators include industrial structures, primary industry output values, demographic trends, political factors, public funds, and public satisfaction, which primarily consider the social and cultural background, subjects involved, and social benefits brought about by the upgrade to green industries. Natural ecological indicators include related indicators, such as the climatic characteristics and biodiversity of Poyang Lake, and pay more attention to the possible ecological, economic, and social benefits as well as the possible external effects brought by wetland protection, thereby reflecting the concept of green development. Concurrently, we need to pay attention to the degree of coupling of the system indicators to characterise the coordination of the development of the social system and the ecosystem.

## 4. Discussion

Ostrom argues that different institutional arrangements will enhance or hinder the mechanisms of cooperation between individuals, and social capital could fully exploit the subjectivity of individual autonomy [26,27,28]. Therefore, based on the theoretical background and the above research results, this paper discusses the factors affecting the relationship between ecological environmental protection and economic development of Poyang Lake and analyses the action plans that could make an organic connection between ecological environmental protection and green development.

### 4.1. Analysis of Influencing Factors

#### 4.1.1. Insufficient Investment in Rural Areas in the Lakeside Area

Although public finance investment in agriculture, rural areas, and farmers in China has continuously increased in recent years, there is still a large financial gap between the investments made and the requirements for achieving regional green development goals. Particularly, in the context of the current slowdown in macroeconomic growth, the investments that can be raised are more limited, and there are several shortcomings in the three cities of the Poyang Lake district [46] that need to be addressed; additionally, limited capital investment increases the challenge of balancing regional development.

Though agricultural pollution discharges in the lake area are large, capital investment is small. Compared to investments on industrial enterprises, the funds for pollution control and environmental management capacity building in rural areas from fiscal channels are relatively limited. In terms of policy support, capital investment and capacity building, the management of pollution from agricultural sources has not received as much attention as that from industrial sources.

In 2018, Jiangxi province received CNY 50 million as wetland protection subsidies from the central government. The scope of wetland compensation has increased from three counties (cities and districts) to 10 counties (cities and districts), CNY 20 million as central wetland compensation funds have been obtained, and 266 community ecological restoration and environmental remediation projects have been implemented, which can benefit the Poyang Lake district and its population of 140,000 people (data compiled according to “Jiangxi Statistical Yearbook 2019”.).

#### 4.1.2. Contradictions between Protection and Development of Lakeside Area

To effectively alleviate the decline of the biological resources of the Yangtze River and its biodiversity, the Jiangxi Aquatic Biological Reserve has implemented a long-term ban on fishing. The productive fishing of natural fishery resources in the Jiangxi section of Yangtze River and Poyang Lake has been completely banned for up to 10 years from 1 January 2020. All aquatic life reserves, the main stream of the Yangtze River Jiangxi, and Poyang Lake are covered under this ban (http://jx.people.com.cn/n2/2019/0826/c186330-33286892.html (accessed on 31 December 2019)).

Large-scale fishing still happens in the Poyang Lake area. According to statistics, many fishing villages have been affected by the fishing ban; among them, there are more than 300 traditional fishing villages. More than 25,000 fishing households are present in the entire lake area, and the total fishery population is approximately 150,000 [47]; of this total population, the majority are couples who fish together. Only a small population of young fishermen exists, as the younger generation either go to school or choose other occupations; the average age of the fishing population is high. The fishing ban, older age, narrow information channels for re-employment, lack of skills other than fishing, and low level of education are the barriers for the sustainable livelihood of the fishing population [48]. Along with the implementation of ecological protection policies such as the ban on fishing, it is also important to address issues such as ensuring the livelihood of fishermen after the ban, smooth transition from production to business, and providing local residents new livelihoods through ecological industries.

#### 4.1.3. Difficulty in Implementing Grassroots Policies

Whether it is environmental protection or green development, the ecological environment is both a foundation and an opportunity for development. The effective implementation of conservation policies determines whether the two strategies can be linked [49]. In the survey, it was found that due to the weak grassroots implementation, lack of technology, and system defects, some environmental protection policies have produced “prohibition instead of governance” and “excessive law enforcement” phenomena in their implementation. We consider that there has been poor regulation of the ecological damage caused by regional economic development compared to the regulation of individual resident behaviour. These phenomena are against the interests of residents and the protection of the ecological environment; they weaken the willingness of the residents of the lake area to protect the environment and intensify conflicts at the grassroots level.

#### 4.1.4. Outflow of Rural Labour in Poyang Lake Area

With the acceleration of urbanisation and the difference in economic development levels between urban and rural areas, a large number of young and middle-aged labourers have migrated to first-tier cities such as Shanghai and Guangzhou, and “hollow villages” have appeared in many areas of Poyang Lake. According to the sixth national census, among the total permanent population of Jiangxi province, the population aged 65 and above accounted for 7.60% of the total (data source: bulletin of the sixth national census of Jiangxi province in 2010.). A high percentage of elderly people reside in the rural areas. Related research indicates that the rural population is expected to decrease from the current 600 million to 450 million in 2035, which means that the process of urbanisation is far from over [50] and the population aging problem will continue to constrain the development of Poyang Lake for a long time.

The loss of rural population from the lakeside area will inevitably cause serious contradictions between the demand and supply of labour with modern agricultural skills. The elderly rural population is more dependent on natural resources and traditional and extensive agricultural and forestry management, causing tremendous pressure on the ecological environment because this population cannot undertake activities with high returns [51]. If no practical measures are taken, the ongoing mass exodus of labour will lead to a labour shortage crisis and a lack of successors, adversely affecting the organic convergence of ecological environmental protection and green development in rural areas. This will inhibit the possibility of a virtuous cycle of green development in rural areas, making it more difficult to implement the strategy of rural revitalisation, thereby causing a series of social and environmental problems [52].

### 4.2. Action Scenario Setting

Human activities are the primary cause of ecological environment deterioration in the Poyang Lake area. To achieve the organic integration between ecological protection and green development, it is necessary to achieve a harmonious symbiosis between man and nature. Therefore, the implementation of ecological environment protection and green development in the Poyang Lake area will inevitably involve many stakeholders, which is a typical social-ecological system case. Every protection project and development project implementations in the important ecological protection area of the Poyang Lake district should systematically analyse the problems and challenges of the economic, social, and ecological environment of the region. Additionally, the projects must mobilise all participants, improve the ecological governance system, monitor the status of and dynamic changes in Poyang Lake resources [53], environment, and ecosystems, and conduct a dynamic evaluation of their effectiveness to ensure the coordinated development of the ecological environment and social economy in the region.

#### 4.2.1. Ecological Environmental Protection Projects

In the Poyang Lake district, solving the outstanding ecological and environmental problems should be considered as a priority; further, the environmental protection responsibility system should be fully implemented, supervision and law enforcement of environmental protection should be increased, comprehensive management of outstanding environmental problems should be strengthened, and people’s expectations of a good living environment must be met. Based on our study, we deduced the following information:

(1) Ecological compensation and implementing the return of farmland to wetland is crucial. After the 1998 flood in the Yangtze River basin, according to the plan of the “project of returning farmland to wetland” and the actual situation of Poyang Lake, it was necessary for the farmland surrounding the lake to be completely returned to wetland to increase the flood storage capacity and enhance the wetland ecosystem service function of Poyang Lake. Hence, farming and livestock breeding in the dike region was banned [54]. Therefore, the key to implementing the policy of returning farmland to wetlands and providing ecological compensation lies in scientifically determining the compensation standard and mode of compensation to effectively increase the enthusiasm of farmers to participate in the transformation of farmland to wetlands [55]. As a micro-decision-making body for wetland protection, the farmers’ willingness to transform farmland to wetland is the basic premise and key to actively promote wetland protection [56].
(2) Wetland ecological restoration management restores the wetland plant communities at a large scale and rewilds wetlands with native species, such as apex predators, to rebuild the balanced ecosystem in large areas of permanent and intermittent wetlands in the Poyang Lake area. In the process of ecological restoration and management, we can consider adding value to macrobenthos to quickly restore the health and stability of the ecosystem, improve the quality of the ecosystem, and enhance ecosystem services [57]. Developing and adopting adequate policy instruments are crucial for reversing the loss of wetland fauna (caused by overfishing and poaching) and exploitation and trade of threatened species, so as to conserve biodiversity and aid in restoration activities [58]. In addition, we proposed lakeshore naturalisation by considering the topographic features of Poyang Lake, reintegrating waterways and bays through wetland systems, promoting habitat restoration, improving water quality, and strengthening flood control mechanisms.
(3) A long-term mechanism for cross-basin protection and pollution prevention and control should be established. In 2018, in Jiangxi province, 1.33 km^2^ of farmland was transformed to wetlands; a total of 102 new wetland protection sites were added, including 97 wetland protection communities and five water source protection sites; 131 km^2^ of area was newly developed to wetlands; the wetland protection rate increased by 1.45%. However, the influence of administrative division makes it difficult to realise the protection of the whole basin of Poyang Lake. In the future, it will be necessary to protect the Poyang Lake wetlands and cooperate with the cities of Nanchang, Jiujiang, and Shangrao as a whole to strive for environment policy-related, project implementation-related, and financial support. There have been studies that have concluded that water resources governance could be managed through a river chief system [59]. We further consider that through four levels of cooperation (city, county, township, and village), the individual protection measures such as the water quality, flood control, and drainage will be combined and upgraded to a long-term pollution prevention and control mechanism covering rivers and lakes, water function areas, and water ecology for the complete management of the Yangtze River, Poyang Lake, and other tributaries. We also recommend creating a benign ecological cycle system, harmonisation across jurisdiction [60], and an ecological safety barrier in the wetlands of Poyang Lake to provide an ecological environment that will ensure green development.

#### 4.2.2. Ecological Priority to Green Development

It will be more beneficial to adopt a new method for coordinated development of ecology and economy and focus on transforming the green ecological advantages into economic development advantages to enhance the ecological wellbeing of the people. Based on our study, we recommend the following points:

(1) We suggest establishing a Poyang Lake National Park. By breaking the boundaries of administrative regions and industries and coordinating natural resources and ecological management within the Poyang Lake region, a National Park must be built covering the upstream and downstream areas of Poyang Lake. In the national park, unified restoration and protection of various ecosystems of mountains, rivers, and lakes can be implemented, along with in situ monitoring and protection of rare and endangered wild animals and plants, such as migratory birds and large aquatic creatures. The areas with unique natural landscapes and rich biodiversity can be protected, and green development of the Poyang Lake basin [61] can be promoted to shift the focus from a “resource economy” to an “ecological economy” and realise the harmonious coexistence between man and nature.
(2) Scientific and technological support systems for coordinated development can be improved using big data, 3S, the Internet of Things, and other information technologies; a dynamic monitoring system is being built for the internal features, which includes mountains, water, forest, farmland, lake, and grass to strengthen the dynamic monitoring capacity of the Poyang Lake ecosystem. Scientific research must be encouraged to assess the ecological health of wetlands and ecosystem service functions and the impact of industrial development on the environment within the ecological economic zone. These technical solutions should also be applied to other ecologically fragile areas. This is because only with a clear understanding of the basics can these areas be better equipped to formulate conservation and development policies.
(3) Promoting the service industry with green development is crucial. The Poyang Lake area is rich in tourism resources, complete in various tourism elements, and has a strong tourism reception capacity, which is the basis for the development of the modern service industry. First, we need to integrate the tourism resources in nature (including humanities, health, and wellness) from Nanchang, Jiujiang, and Shangrao as well as the surrounding cities. Attention must be given to designing cross-regional religious culture boutique theme tourist routes, with Longhu Mountain (Longhu Mountain is located 20 km southwest of Yingtan city, Jiangxi province, and is the birthplace of Chinese Taoism, a world natural heritage and a world geological park) and Sanqing Mountain (Sanqing Mountain is located at the junction of Yushan county and Dexing city in Shangrao city, Jiangxi province, China; Sanqing Mountain is a famous Taoist mountain, a world natural heritage site, a world geological park, and the source of the Xinjiang River) as the core, to enhance the influence of the all-for-one tourism concept in the Poyang Lake region at regional and global levels. Second, on the basis of the ecological functional zoning of the Poyang Lake basin, a rational division of labour between upstream and downstream counties will be carried out [62], including wetland ecotourism and recreation, ecological agriculture and forestry, fishery breeding, and ecological characteristic industries. Both models, if they could be adopted in other developing countries, would reduce direct damage to local natural resources and would also develop local economies.
(4) We propose developing modern agriculture and building “beautiful villages” in the region. First, it is important promote modern agriculture, devise and promote green technologies, and adopt renewable energy sources, such as photovoltaic (PV) and wind power, which do not adversely impact wetlands [63]. Second, it is important to optimise the spatial layout of agriculture and build a national supply base (for bulk high-quality green agricultural products) and an aquaculture base. Third, production and transaction costs should be lowered through collective cooperative organisations to reduce the contradiction between the decentralised operation of small farmers and the standardised development of agriculture at a large scale. Fourth, we must improve the condition of rural roads, ensure the safety of water sources, and improve rural infrastructure. Implementation of agricultural modernisation in the Poyang Lake area not only increases the income of farmers, but also encourages young people to return to the villages. Additionally, competitive green agricultural industry chains of grains, fruits and vegetables, and fish and shrimp can be maintained along with the natural environment and “beautiful villages”. This approach is in line with the Chinese government’s commitment to the UN sustainable development goals for 2030.

#### 4.2.3. Organisation and Implementation of Organic Linking between Ecological Protection and Green Development

On the basis of field investigation and understanding of the ecological protection system and mechanism of Poyang Lake in Jiangxi, it is necessary to re-examine the ecological protection and economic development measures implemented in Poyang Lake in the past two decades and analyse the key problems of its incompatibility. It is best to clarify the reformed direction and mechanism of the system and put forward a protection and development system guarantee.

(1) The supply of a new system is the core element for achieving organic convergence between ecological protection and green development. Some studies have concluded that one of the important advantages of China’s national governance system is that, when faced with major problems, it provides new institutional supplies to cope with rapid changes in the ecological environment and socio-economic conditions [64,65]. On this basis, we believe that for the ecological protection and green development of Poyang Lake, it is extremely important to introduce a new system, improve the ecological protection policy, improve the capacity building of the grassroots staff, and encourage social subjects in the lakeside area to participate in environmental protection and green development.
(2) Credible commitment is a key condition for the system to operate. As the ecological governance system in China is dominated by the administrative authorities, the provincial government of Jiangxi province can act as the leading authority to define the ownership of the Poyang Lake wetland as public property and identify the limits of its resources. In addition, the use of performance appraisal and environmental monitoring enables the effective supervision of the involved entities, which avoids the recurrence of excessive law enforcement. A commitment system covering provinces, cities, counties, and townships can be formed to determine the boundaries of authority and responsibility of units at all levels, solve the problems in the implementation of policies at the grassroots level, and lay the foundation for the change of ecological governance in the Poyang Lake basin.
(3) Implementing a dynamic assessment system is suggested to strengthen assessment and supervision. In the four-level administrative system of the Chinese Government, the central government and higher-level governments always hold the power of supervision and inspection [65]. Therefore, the utilisation of performance appraisal and environmental monitoring will enable the effective monitoring of participating subjects by higher levels of government. When the ecological environment generates externalities, as environmental performance is integrated into the hierarchical system of government operation at all levels, the monitoring mechanism within the administrative system can also work effectively, despite the specificity of the ecological and environmental problems in the large-scale space and time of the Poyang Lake basin in Jiangxi province [64]. Intergovernmental management of ecological problems through administrative subcontracting can solve the problem of ensuring regional development through ecological protection.
(4) We recommend estimating capital investment and formulating a fund-raising plan, including increasing and implementing government funding at all levels. This will help in dynamically adjusting the funding to favour urgent projects. The funds can first be dissolved to the three municipalities in the lakeside regions, and after achieving results in utilising the funds aids to strengthen the responsibilities of local entities, allocated to the Poyang Lake Ecological Economic Zone and the entire Jiangxi province. The economy of residents of the lake area should be decentralised to reduce wealth inequality by assisting more people in relative poverty and it must be ensured that prices, taxation, and incentive systems take into account the real costs imposed by consumption patterns on wetlands [16]. Further, we can accelerate the promotion of the market-oriented allocation of resources and environment in the Poyang Lake basin, make full use of market mechanisms to broaden financing channels, and ensure the implementation of ecological environmental protection and green development in the lake area.
(5) In addition, public participation should be encouraged. Past models of conservation and development have produced a large number of experienced individuals with a wide range of skills and experience, which is an advantage that is not available worldwide. Outdoor wetland education should be increased for children and adults, along with the involvement of society, including local and indigenous communities, for the management of wetlands in the future. The role of individual residents in solving the multitasking problems in the protection and development of Poyang Lake is very important because it requires rich accumulation of human capital.

## 5. Conclusions

Therefore, at the theoretical level, promoting the coordination of protection and development in Poyang Lake basin is not simply to restrict the development of economy in the region, but more to promote the transformation of economic development mode to industrial ecology and ecological industrialization, focus on the development of social public utility fields and promote the modernization level of the ecological governance system and the improvement of governance capacity in Jiangxi province. At the practical level, the process of promoting the organic connection between ecological environmental protection and green development should avoid simple policy orientation and realistic orientation and needs to take the whole Poyang Lake ecosystem as a whole, scientifically plan the ecological protection measures and economic development policies, narrow the gap between urban and rural areas, and reduce the destruction of the ecological environment.

Previous studies using the SES framework were limited by their scales and did not focus enough on the external political, economic and social environment, but more on analysing the relationship between the internal state variables of the system. This study, however, expands on previous research by including the external environment as an object of study and paying more attention to the influence of the governance system on the relationship between ecological protection and green development. Additionally, wetland ecosystems are very special ecosystems, and it is relatively rare to study the relationship between wetland conservation and economic development, especially using the SES framework. Therefore, this study also enriches the scope of application of the SES framework. The analysis method for the social-ecological system of Poyang Lake portrays a new paradigm for analysing complex governance systems. However, in the case study, the interaction process between actors (As) was not analysed in depth. This is because the design of the SES framework (I and O) does not reflect a few typical characteristics of the protection and development efforts. Future research based on this work should apply our framework to a more suitable analytical framework for China’s national conditions, which will allow policy-makers and researchers to conduct more discussions, analyses, and practices in terms of policy discourse, policy tools, working mechanisms, and green development strategies. This will promote the early improvement and implementation of ecological environmental protection and green development through the integration of practical policy implementation and academic discussions.

## Figures and Tables

**Table 1:** Analysis variable table of organic integration between ecological environment protection and green development in Poyang Lake wetland.

Related Ecosystem (ECO)	Resource System (RS)	Actor (A)
ECO1 Climate characteristics * -ECO11 Hydrothermal conditions --ECO111 Average annual temperature --ECO112 Average annual precipitation --ECO113 Annual relative humidity -ECO12 Physical geography --ECO121 Altitude ECO2 Type of pollution * ECO3 Flow of resources *	RS1 Resource Type * -RS11 Types of wetland resources RS2 Clear resource boundaries RS3 Size of the resource system* -RS31 Fish farming area -RS32 Wetland area per capita RS4 Output of resource system* -RS41 Fishery aquaculture yield RS5 Balance RS6 System dynamic predictability RS7 Position	A1 Number of relevant actors A2 Socio-economic attributes A3 History or past experience A4 Social status A5 leadership A6 Social capital A7 Social Ecosystem Concept A8 Dependence on resources A9 Available technology
**Social, Economic, Political Setting (S)**	**Resource Unit (RU)**	**Interaction (I)**
S1 Economic development * -S11 Regional economic level --S111 Gross Domestic Product (GDP) --S112 Primary industry output value --S113 Revenue --S114 Total grain output --S115 Urban residents’ income --S116 Rural residents’ income S2 Demographic trends * -S21 Total population --S211 Agriculture and forestry population --S212 Fishery population S3 Political stability * -S31 Central government attention --S311 Impact on the Yangtze River -S32 Local government attention S4 Other governance systems S5 Market S6 Technology	RU1 Resource unit mobility RU2 Renewal or growth rate RU3 Interaction between resource units RU4 Economic Value RU5 Scale RU6 Notable mark RU7 Temporal and spatial distribution	I1 The harvest volume of different users I2 Information sharing among us-ers I3 Negotiation process I4 Conflicts between users I5 Investment Activities I6 Self-organising action
	**Governance System (GS)**	**Outcome (O)**
	GS1 Government organisation GS2 Non-Governmental Organisations (NGO) GS3 Property structure GS4 Implementation plan (province, city) -GS41 Ecological migration (local funds) GS5 Collective selection rules (province, city) -GS51 Local financial support GS6 Protection policy (national) * -GS61 Ecological protection project and policy --GS611 Returning farmland to wetland (returning farmland to lake) Project --GS612 Wetland ecological compensation mechanism --GS613 No fishing season subsidy GS7 Green development project GS8 Monitoring and inspection process	O1 Social performance evaluation O2 ecological performance evaluation O3 Other SES system externalities

Variables in the table are sorted and revised based on the results of McGinnis and Ostrom (2014) [30]. Note: Variables marked with * are heterogeneous variables that affect the organic linking of ecological protection and green development.

**Table 2:** Comparison of Poyang Lake wetland Ecosystem (ECO).

Variable	Nanchang City	Jiujiang City	Shangrao City
ECO1 Climate characteristics (2017)			
-ECO11 Hydrothermal conditions			
--ECO111 Annual average temperature (°C)	19	17	19
--ECO112 Annual precipitation (mm)	1611	1967	1866
--ECO113 Annual relative humidity (%)	73	81	73
ECO2 Type of pollution (2017)			
-ECO21 Total industrial wastewater discharge (10 kilotons)	3861	7904	6318
-ECO22 Total industrial emissions (TMC)	1876	2601	1317

Data compiled according to “Jiangxi Statistical Yearbook 2018”. Note: - at secondary variable, -- at explanatory variable, respectively.

**Table 3:** Comparison of Poyang Lake wetland social, economic, political setting (S).

Variable	Nanchang City	Jiujiang City	Shangrao City
S1 Economic Development (2017)			
-S11 Regional economic level			
--S111 Regional GDP (100 million yuan)	5003	2414	2024
--S112 Primary industry (100 million yuan)	192	193	247
--S113 Total fiscal revenue (100 million yuan)	783	461	319
--S114 Total grain output (10,000 tons)	238	173	352
--S115 Per capita disposable income of urban residents (yuan)	37,675	32,592	31,853
--S116 Per capita disposable income of rural residents (yuan)	16,364	13,303	12,174
S2 Demographic trends (2017)			
-S21 Total population (ten thousand people)	546	487	678
S3 Political factors			
-S31 Central importance	High	High	Middle
--S311 Impact on Yangtze River basin	High	High	Middle

Data compiled according to “Jiangxi Statistical Yearbook 2018”. Note: - at secondary variable, -- at explanatory variable, respectively.

**Table 4:** Comparison of Poyang Lake wetland resource system (RS).

Variable	Nanchang City	Jiujiang City	Shangrao City
RS1 resource type:			
-RS11 wetland resource type:			
River wetland (ha)	33,842	40,824	47,083
Lake wetland (ha)	95,603	174,597	96,927
Swamp (ha)	9452	1766	14,156
Artificial wetland (ha)	14,375	47,632	33,065
Total (ha)	153,272	264,819	191,231
RS3 Scale of Resource System:			
-RS31 Fish farming area (ha)	53,605	78,001	79,530
RS4 Output of resource system:			
-RS41 Fishery aquaculture yield (kg/ha)	6328	4836	5580

Data compiled according to “Jiangxi Statistical Yearbook 2018”. Note: - at secondary variable, respectively.

**Table 5:** Comparison of Poyang Lake wetland governance system (GS).

Variable	Nanchang City	Jiujiang City	Shangrao City
GS1 Government organisation	YES	YES	YES
GS2 Non-Governmental organisations (NGO)	YES	YES	YES
GS5 Collective selection rules (province, city)			
-GS51 Local financial support	YES	YES	YES
GS6 Protection policy (national) *	YES	YES	NA
-GS61 Ecological protection project and policy	YES	YES	YES
--GS611 Returning farmland to wetland (returning farmland to lake) project	YES	YES	YES
--GS612 Wetland ecological compensation mechanism	YES	YES	NA
--GS613 No fishing season subsidy	YES	YES	YES
GS7 Green development project	YES	YES	YES

Note: - at secondary variable, -- at explanatory variable, Variables marked with * are heterogeneous variables that affect the organic linking of ecological protection and green development, respectively.

## Data Availability

Data available in a publicly accessible repository that does not issue DOIs.

## Appendix A

**Table A1:** Sources of data in the article.

Variable	Data Source
Annual average temperature	Jiangxi Statistical Yearbook 2018
Annual precipitation	Jiangxi Statistical Yearbook 2018
Annual relative humidity	Jiangxi Statistical Yearbook 2018
Type of pollution	Jiangxi Statistical Yearbook 2018
Total industrial wastewater discharge	Jiangxi Statistical Yearbook 2018
Total industrial emissions	Jiangxi Statistical Yearbook 2018
Average water level	2015 Poyang Lake Scientific Research Report
Average annual siltation of sediment	Chen Minkun, Xu Xibao (2021) as [41]
Number of phytoplankton species	Chen Minkun, Xu Xibao (2021) as [41]
Total storage capacity	2015 Poyang Lake Scientific Research Report
Population that faces poverty	http://www.gov.cn/xinwen/2020-04/27/content_5506419.htm
Regional GDP	Jiangxi Statistical Yearbook 2018
Primary industry	Jiangxi Statistical Yearbook 2018
Total fiscal revenue	Jiangxi Statistical Yearbook 2018
Total grain output	Jiangxi Statistical Yearbook 2018
Per capita disposable income of urban residents	Jiangxi Statistical Yearbook 2018
Per capita disposable income of rural residents	Jiangxi Statistical Yearbook 2018
River wetland	Jiangxi Statistical Yearbook 2018
Lake wetland	Jiangxi Statistical Yearbook 2018
Swamp	Jiangxi Statistical Yearbook 2018
Artificial wetland	Jiangxi Statistical Yearbook 2018
Fish farming area	Jiangxi Statistical Yearbook 2018
Fishery aquaculture yield	Jiangxi Statistical Yearbook 2018

## References

[1] Yanli L., Sibao D., Rongcheng W., Yu W. (2010). Sustainable Development of Vulnerable Ecological Regions under the Perspective of Regional Externalities. China Popul. Resour. Environ..

[2] Yahua W., Rui G., Qingguo M. (2016). Crisis and Response of Chinese Rural Public Affair Governance. J. Tsinghua Univ..

[3] Zehong L., Yongqing B., Jiulin S., Suocheng D., Jingnan L. (2019). Ecological Civilization Construction in Ecologically Fragile Poverty-Stricken Areas in Western China. Strateg. Study CAE.

[4] Water C.D. (2019). Health, and the Economic-Canada Green Plan. Res. J. Water Pollut. Control Fed..

[5] Pearce D. (1990). Environmentalism and the Green Economy. Environ. Plan. A.

[6] Duthie D. (2001). How to Grow a Green Economy. New Sci..

[7] Rojšek I. (2001). From Red to Green: Towards the Environmental Management in the Country in Transition. J. Bus. Ethics.

[8] White D.F. (2002). A Green Industrial Revolution? Sustainable Technological Innovation in a Global Age. Environ. Pol..

[9] Brown E., Cloke J., Gent D., Johnson P.H., Hill C. (2014). Green Growth or Ecological Commodification: Debating the Green Economy in the Global South. Geogr. Ann. B.

[10] Schulz C., Bailey I. (2014). The Green Economy and Post-Growth Regimes: Opportunities and Challenges for Economic Geography. Geogr. Ann. Ser. B Hum. Geogr..

[11] Yando E.S., Osland M.J., Jones S.F., Hester M.W. (2019). Jump-Starting Coastal Wetland Restoration: A Comparison of Marsh and Mangrove Foundation Species. Restor. Ecol..

[12] Wenmin H., Guo L., Gao Z., Jia G., Yi L. (2020). Assessment of the Impact of the Poplar Ecological Retreat Project on Water Conservation in the Dongting Lake Wetland Region Using the InVEST Model. Sci. Total Environ..

[13] Mansur A.V., Brondízio E.S., Roy S., Hetrick S., Vogt N.D., Newton A. (2016). An Assessment of Urban Vulnerability in the Amazon Delta and Estuary: A Multi-Criterion Index of Flood Exposure, Socio-Economic Conditions and Infrastructure. Sustain. Sci..

[14] Sebesvari Z., Renaud F.G., Haas S., Tessler Z., Hagenlocher M., Kloos J., Szabo S., Tejedor A., Kuenzer C. (2016). A Review of Vulnerability Indicators for Deltaic Social–Ecological Systems. Sustain. Sci..

[15] Srinivas R., Singh A.P., Dhadse K., Garg C., Deshmukh A. (2018). Sustainable Management of a River Basin by Integrating an Improved Fuzzy Based Hybridized SWOT Model and Geo-Statistical Weighted Thematic Overlay Analysis. J. Hydrol..

[16] Finlayson C.M., Davies G.T., Moomaw W.R., Chmura G.L., Natali S.M., Perry J.E., Roulet N., Sutton-Grier A.E. (2019). The Second Warning to Humanity—Providing a Context for Wetland Management and Policy. Wetlands.

[17] An-gang H.U., Shao-jie Z. (2014). Green Development: Functional Definition, Mechanism Analysis and Development Strategy. China Popul. Resour. Environ..

[18] Xiangmin X.U. (2018). Transcending Sustainable Development View through Green Development Thought and Innovation of Green Legal System. Leg. Forum.

[19] Yu L. (2020). Insisting on High-Quality Ecological Protection of the Yellow River and Promoting High-Quality Green Development of the River Basin. Environ. Prot..

[20] Chengcai T., Wenjing F., Lei Z.H.U. (2014). The Discussion on Development Model of Wetland Ecotourism for Yeya Lake in Beijing. Ecol. Econ..

[21] Wenbiao R. (2019). The Organic Linking of Small-Scale Farmers with Modern Agricultural Development in China: Empirical Evidence, Outstanding Contradictions and Path Choice. China Rural Surv..

[22] Yiqing S., Ming Q., Yahua W. (2020). The Impact of Farmland Transfer on Rural Collective Action under the Scenario of Labor Outmigration:A Research Based on Social-Ecological System (SES)Framework. Manag. World.

[23] Walker B., Holling C.S., Carpenter S.R., Kinzig A.P. (2004). Resilience, Adaptability and Transformability in Social-Ecological Systems. Ecol. Soc..

[24] Folke C., Hahn T., Olsson P., Norberg J. (2005). Adaptive Governance of Social-Ecological Systems. Annu. Rev. Environ. Resour..

[25] Liu J.G., Dietz T., Carpenter S.R., Alberti M., Folke C., Moran E., Pell A.N., Deadman P., Kratz T., Lubchenco J. (2007). Complexity of Coupled Human and Natural Systems. Science.

[26] Ostrom E. (2007). A Diagnostic Approach for Going beyond Panaceas. Proc. Natl. Acad. Sci. USA.

[27] Ostrom E. (2009). A General Framework for Analyzing Sustainability of Social-Ecological Systems. Science.

[28] Ostrom E. (2010). Beyond Markets and States: Polycentric Governance of Complex Economic Systems. Am. Econ. Rev..

[29] Yanmei Y., Yaoben L., Shuchang L., Ming L. (2019). Social-Ecological System (SES) Analysis Framework for Application in Ecological Restoration Engineering of Mountains-Rivers-Forests-Farmlands-Lakes-Grasslands: Utilizing the Source Area of Qiantang River in Zhejiang Province as an Example. Acta Ecol. Sin..

[30] Mcginnis M.D., Ostrom E. (2014). Social-Ecological System Framework: Initial Changes and Continuing Challenges. Ecol. Soc..

[31] Haibo Q., Xingjun R., Yingming L. (2018). Research on Sustainable Grassland Governance Mechanisms in China Based on Social-Ecological System Framework. J. Gansu Admin. Inst..

[32] Shijun M., Rusong W. (1984). The Social-Economic-Natural Complex Ecosystem. Acta Ecol. Sin..

[33] Levin S.A. (2000). Fragile Dominion: Complexity and the Commons.

[34] Leslie H.M., Basurto X., Nenadovic M., Sievanen L., Cavanaugh K.C., Cota-Nieto J.J., Erisman B.E., Finkbeiner E., Hinojosa-Arango G., Moreno-Báez M. (2015). Operationalizing the Social-Ecological Systems Framework to Assess Sustainability. Proc. Natl. Acad. Sci. USA.

[35] Brondizio E.S., Ostrom E., Young O.R. (2009). Connectivity and the Governance of Multilevel Social-Ecological Systems: The Role of Social Capital. Annu. Rev. Environ. Resour..

[36] Basurto X., Gelcich S., Ostrom E. (2013). The Social–Ecological System Framework as a Knowledge Classificatory System for Benthic Small-Scale Fisheries. Glob. Environ. Chang..

[37] Virapongse A., Brooks S., Metcalf E.C., Zedalis M., Gosz J., Kliskey A., Alessa L. (2016). A Social-Ecological Systems Approach for Environmental Management. J. Environ. Manag..

[38] Brondizio E.S., Vogt N.D., Mansur A.V., Anthony E.J., Costa S., Hetrick S. (2016). A Conceptual Framework for Analyzing Deltas as Coupled Social–Ecological Systems: An Example from the Amazon River Delta. Sustain. Sci..

[39] Seeteram N.A., Engel V., Mozumder P. (2018). Implications of a Valuation Study for Ecological and Social Indicators Associated with Everglades Restoration. Sci. Total Environ..

[40] Andrews S.S., Karlen D.L., Cambardella C.A. (2004). The Soil Management Assessment Framework: A Quantitative Soil Quality Evaluation Method. Soil Sci. Soc. Am. J..

[41] Minkun C., Xibao X. (2021). Lake Poyang Ecosystem Services Changes in the Last 30 Years. J. Lake Sci..

[42] Huang Z., Lu L., Jiao G., Jiang J., Ye Q. (2018). Analysis of the Correlations between Environmental Factors and Rare Cranes in the Poyang Lake Region of China. J. Great Lakes Res..

[43] Xiaoming G., Jie G. (2019). How to Effectively Connect Poverty Alleviation and Rural Revitalization Policy Implementation. J. Soc. Theor. Guide.

[44] Zhang Q., Ye X.C., Werner A.D., Li Y., Yao J., Li X., Xu C. (2014). An Investigation of Enhanced Recessions in Poyang Lake: Comparison of Yangtze River and Local Catchment Impacts. J. Hydrol..

[45] Sterling E., Ticktin T., Morgan T.K., Cullman G., Alvira D., Andrade P., Bergamini N., Betley E., Burrows K., Caillon S. (2017). Culturally Grounded Indicators of Resilience in Social-Ecological Systems. J. Environ. Soc..

[46] Shengwei T. (2020). The Organic Integration of Poverty Alleviation and Rural Revitalization Strategies: Goal Orientation, Key Areas and Measures. Chin. Rural Econ..

[47] Jingbo S. (2017). Survival Situation of Fishermen in Poyang Lake and the Difficulties and Countermeasures for Change of Production and Occupation. Jiangxi Fish. Sci. Technol..

[48] Frawley T.H., Crowder L.B., Broad K. (2019). Heterogeneous Perceptions of Social-Ecological Change among Small-Scale Fishermen in the Central Gulf of California: Implications for Adaptive Response. Front. Mar. Sci..

[49] Hu Y., Rao F., Shuqin J. (2019). Eco-Environmental Concerns in the Organic Connection between Poverty Alleviation and Rural Revitalization. Reform.

[50] Xiwen C. (2018). Implementing the Rural Revitalization Strategy and Promoting Agricultural and Rural Modernization. J. China Agric. Univ. Soc. Sci. Ed..

[51] Nguyen T.T., Do T.L., Bühler D., Hartje R., Grote U. (2015). Rural Livelihoods and Environmental Resource Dependence in Cambodia. Ecol. Econ..

[52] Zhang Y.C., Westlund H., Klaesson J. (2020). Report from a Chinese Village 2019: Rural Homestead Transfer and Rural Vitalization. Sustainability.

[53] Feng L., Hu C., Chen X., Cai X., Tian L., Gan W. (2012). Assessment of Inundation Changes of Poyang Lake Using MODIS Observations between 2000 and 2010. Remote Sens. Environ..

[54] Liu Y., Feng J., Zheng-lei X.I.E., Jun-bang W., Shu-hua L.F.Q.I. (2017). Study on Land Reclamation around Poyang Lake in the Abandoned Farmland in the Context of the Policy for Converting Farmland to Lake. China Land Sci..

[55] HongGen Z., HuiZhen J., LanYuan K., Feng W.U. (2015). An Empirical Analysis of Wetland Restoration Compensation Standards Based on Farmers’ WTA: Survey Data from 1009 Farmers in Poyang Lake. Fin. Trade Res..

[56] Kong F., Xiong K., Zhang N. (2014). Determinants of Farmers’ Willingness to Pay and Its Level for Ecological Compensation of Poyang Lake Wetland, China: A Household-Level Survey. Sustainability.

[57] Gann G.D., Mcdonald T., Walder B., Aronson J., Nelson C.R., Jonson J., Hallett J.G., Eisenberg C., Guariguata M.R., Liu J. (2019). International principles and standards for the practice of ecological restoration. Restor. Ecol..

[58] Lepczyk C.A., Aronson M.F.J., Evans K.L., Goddard M.A., Lerman S.B., MacIvor J.S. (2017). Biodiversity in the City: Fundamental Questions for Understanding the Ecology of Urban Green Spaces for Biodiversity Conservation. BioScience.

[59] Wang Y., Chen X., Tortajada C. (2020). River Chief System as a Collaborative Water Governance Approach in China. Int. J. Water Resour. Dev..

[60] Hualin X., Peng W., Hongsheng H. (2013). Ecological Risk Assessment of Land Use Change in the Poyang Lake Eco-Economic Zone, China. Int. J. Environ. Res. Public Health.

[61] Bell-James J., Boardman T., Foster R. (2020). Can’t see the (mangrove) forest for the trees: Trends in the legal and policy recognition of mangrove and coastal wetland ecosystem services in Australia. Ecosyst. Serv..

[62] Zhongyuan Y. (2020). Study on the Optimization of Hainan Regional Governance System Based on Watershed Social Ecosystem. J. Hainan Norm. Univ. Nat. Sci..

[63] Acosta C., Ortega M., Bunsen T., Koirala B.P., Ghorbani A. (2018). Facilitating Energy Transition through Energy Commons: An Application of Socio-Ecological Systems Framework for Integrated Community Energy Systems. Sustainability.

[64] Li X., Yang X., Wei Q., Zhang B. (2019). Authoritarian Environmentalism and Environmental Policy Implementation in China. Resour. Conserv. Recy..

[65] Xueguang Z. (2012). Lian Hong. Modes of Governance in the Chinese Bureaucracy: A “Control Rights” Theory. J. Sociol. Stud..

